
JOURNAL INFORMATION
==============================
NLM Title Abbreviation: Immun Inflamm Dis
Journal ID: Immun Inflamm Dis
NLM Journal ID: 101635460
Journal ID: IID3

Immunity, Inflammation and Disease

EISSN: 2050-4527
Publisher: Wiley

ARTICLE INFORMATION
==============================
PMCID: PMC8127561
DOI: 10.1002/iid3.310
Article ID: IID3310
Article version: 1
Subjects: Issue Information, Issue Information

Issue Information

Electronic publication date: 2021 May 17
Publication date: 2021 Jun
Volume: 9
Issue: 2
First page: 329
Last page: 330
Copyright: © 2021 The Authors. Immunity, Inflammation and Disease published by John Wiley & Sons Ltd.
License: This is an open access article under the terms of the http://creativecommons.org/licenses/by/4.0/ License, which permits use, distribution and reproduction in any medium, provided the original work is properly cited.
License URL: https://creativecommons.org/licenses/by/4.0/

Figure count: 0
Table count: 0
Page count: 2

source-schema-version-number: 2.0
cover-date: June 2021
details-of-publishers-convertor: Converter:WILEY_ML3GV2_TO_JATSPMC version:6.0.2 mode:remove_FC converted:17.05.2021

==============================
Immunity, Inflammation and Disease
Open Access

### Aims and Scope

*Immunity, Inflammation and Disease* is a peer-reviewed, open access, interdisciplinary journal providing rapid publication of cutting-edge research across the broad field of immunology. *Immunity, Inflammation and Disease* gives rapid consideration to papers in all areas of clinical and basic research. The journal welcomes original work that enhances the understanding of immunology in areas including cellular and molecular immunology, clinical immunology, allergy, immunochemistry, immunogenetics, cancer immunology, and many others.

Original research articles, methods papers, editorials and commentaries are published. Original research papers must report well-conducted research with conclusions supported by the data presented in the paper. *Immunity, Inflammation and Disease* considers for publication both papers submitted directly to the journal and those referred from a select group of prestigious journals published by Wiley Blackwell. For further information, please refer to our Author Guidelines available at http://onlinelibrary.wiley.com/journal/10.1002/(ISSN)2050-4527/homepage/ForAuthors.html

### Open Access and Copyright

*Immunity, Inflammation and Disease* is a Wiley Open Access journal, one of a series of peer-reviewed titles publishing quality research with speed and efficiency. For further information, please visit the Wiley Open Access website at http://www.wileyopenaccess.com/view/index.html

All articles published by *Immunity, Inflammation and Disease* are fully open access: immediately freely available to read, download and share. Articles are published under the terms of the Creative Commons Attribution License which permits use, distribution and reproduction in any medium, provided the original work is properly cited.

Copyright on any research article in a journal published by *Immunity, Inflammation and Disease* is retained by the author(s). Authors grant Wiley a licence to publish the article and identify itself as the original publisher. Authors also grant any third party the right to use the article freely as long as its integrity is maintained and its original authors, citation details and publisher are identified. Further information about open access license and copyright can be found in this website: http://www.wileyopenaccess.com/details/content/12f25db4c87/Copyright--License.html

### Disclaimer

The Publisher and Editors cannot be held responsible for errors or any consequences arising from the use of information contained in this journal; the views and opinions expressed do not necessarily reflect those of the Publisher and Editors, neither does the publication of advertisements constitute any endorsements by the Publisher and Editors of the products advertised.

Wiley Open Access articles posted to repositories or websites are without warranty from Wiley of any kind, either express or implied, including, but not limited to, warranties of merchantability, fitness for a particular purpose, or non-infringement. To the fullest extent permitted by law Wiley disclaims all liability for any loss or damage arising out of, or in connection with, the use of or inability to use the content.

### Editor-in-Chief

Marc Veldhoen, PhD

*Principal Investigator*
*Instituto de Medicina Molecular (iMM)*
*Lisbon, Portugal*
*Email: Marc Veldhoen (marc.veldhoen@medicina.ulisboa.pt)*

*Address correspondence to the Editorial Office:*
*Email: IIDeditorial@wiley.com*

### Editorial Board

Omid Akbari, PhD
*Professor of Molecular Microbiology & Immunology*
*Keck School of Medicine*
*University of Southern California, USA*

Matthew Buckland, MBBS MSc PhD FRCP FRCPath
*Consultant and Honorary Senior Lecturer*
*Institute of Child Health*
*University College London, UK*
*Consultant Immunologist*
*Barts Health NHS Trust, UK*

Niels Olsen Saraiva Câmara, PhD
*Professor of Department of Immunology*
*Institute of Biomedical Sciences*
*University of São Paulo, Brazil*

Mark Coles, PhD, FRSB
*Director of Graduate Studies*
*Kennedy Institute of Rheumatology*
*University of Oxford, UK*

Anselm Enders, MD
*Senior Research Fellow*
*The Australian National University, Australia*

JieRuo Gu, MD
*Director*
*Department of Rheumatology*
*Third Affiliated Hospital of Sun Yat-sen University, Guangzhou, China*

Sofia Grigoriadou, MD
*Consultant Immunologist*
*Pathology & Pharmacy Building*
*The Royal London Hospital, UK*

Jochen Huehn, MD
*Department of Experimental Immunology*
*Helmholtz Centre for Infection Research, Germany*

Stéphanie Hugues, PhD
*Assistant professor*
*Department of Pathology and Immunology*
*University of Geneva, Switzerland*

Igor Jurak, PhD
*Assistant professor*
*Department of Biotechnology*
*University of Rijeka, Croatia*

Ludger Klein, PhD, MD
*Institute for Immunology*
*Ludwig Maximilian University of Munich, Germany*

Jun Kunisawa, PhD
*Director of Center for Vaccine and Adjuvant Research*
*National Institute of Biomedical Innovation, Japan*

Arian Laurence, PhD
*National Institute of Arthritis and Musculoskeletal and Skin Diseases USA*

Kathy McCoy, PhD, MD
*Director*
*Department of Physiology and Pharmacology*
*International Microbiome Centre (IMC), Canada*

Hlumani Ndlovu, PhD
*Department of Integrative Biomedical Sciences*
*University of Cape Town, South Africa*

Faith Osier, PhD
*Clinical Research Fellow and Group Leader*
*Centre for Geographic Medicine Research, Coast (KEMRI-CGMRC)*
*Kenya Medical Research Institute, Kenya*

Osamu Takeuchi, PhD, MD
*Professor*
*Kyoto University, Japan*

Hai Qi, PhD, MD
*Professor*
*School of Medicine*
*Tsinghua University, China*

Timothy Radstake, PhD, MD
*Professor of Translational Immunology*
*University Medical Center Utrecht, Netherlands*

Cintia Roodveldt, PhD
*Researcher*
*Andalusian Centre for Molecular Biology and Regenerative Medicine (CABIMER), Spain*

Margarida Saraiva, PhD
*Group leader*
*Institute for Molecular and Cell Biology, Portugal*

Bruno Silva-Santos, PhD
*Professor of Immunology*
*Faculty of Medicine*
*University of Lisbon, Portugal*

Nan Shen, Phd, MD
*Professor of Medicine and Director*
*Shanghai Institute of Rheumatology*
*Ren Ji Hospital Shanghai Jiao Tong University School of Medicine, China*

Mark Travis, PhD
*Professor of Immunology*
*School of Biological Sciences*
*University of Manchester, UK*

David Withers, PhD
*Wellcome Trust funded Research Fellow*
*University of Birmingham, UK*
